# The Re-Establishment of Desiccation Tolerance in Germinated *Arabidopsis thaliana* Seeds and Its Associated Transcriptome

**DOI:** 10.1371/journal.pone.0029123

**Published:** 2011-12-14

**Authors:** Julio Maia, Bas J. W. Dekkers, Nicholas J. Provart, Wilco Ligterink, Henk W. M. Hilhorst

**Affiliations:** 1 Wageningen Seed Lab, Laboratory of Plant Physiology, Wageningen University, Wageningen, The Netherlands; 2 Department of Molecular Plant Physiology, Utrecht University, Utrecht, The Netherlands; 3 Department of Cell and Systems Biology/Centre for the Analysis of Genome Evolution and Function, University of Toronto, Toronto, Canada; Instituto de Biología Molecular y Celular de Plantas, Spain

## Abstract

The combination of robust physiological models with “omics” studies holds promise for the discovery of genes and pathways linked to how organisms deal with drying. Here we used a transcriptomics approach in combination with an *in vivo* physiological model of re-establishment of desiccation tolerance (DT) in *Arabidopsis thaliana* seeds. We show that the incubation of desiccation sensitive (DS) germinated Arabidopsis seeds in a polyethylene glycol (PEG) solution re-induces the mechanisms necessary for expression of DT. Based on a SNP-tile array gene expression profile, our data indicates that the re-establishment of DT, in this system, is related to a programmed reversion from a metabolic active to a quiescent state similar to prior to germination. Our findings show that transcripts of germinated seeds after the PEG-treatment are dominated by those encoding LEA, seed storage and dormancy related proteins. On the other hand, a massive repression of genes belonging to many other classes such as photosynthesis, cell wall modification and energy metabolism occurs in parallel. Furthermore, comparison with a similar system for *Medicago truncatula* reveals a significant overlap between the two transcriptomes. Such overlap may highlight core mechanisms and key regulators of the trait DT. Taking into account the availability of the many genetic and molecular resources for Arabidopsis, the described system may prove useful for unraveling DT in higher plants.

## Introduction

Desiccation tolerance (DT), or anhydrobiosis, can be conceptually defined as the ability to survive, by reversible cessation of metabolism, the removal of almost all cellular free water when in equilibrium with moderately dry air and resume normal function when re-hydrated [1]. By definition, desiccation tolerance is the ability of living organisms to deal with water losses below 0.1 g H_2_O g^−1^ dry weight and survive the re-hydration process without permanent damage [2].

Orthodox seeds acquire DT during development, concomitantly with a myriad of other processes like cell proliferation, reserve deposition, developmental arrest and maturation drying [3]. DT in (orthodox) seeds is based on a range of relatively complex protection mechanisms that accompany dehydration [4]. A strong correlation between protective mechanisms activated during dehydration, such as the accumulation of Late embryogenesis abundant (LEA) proteins and dehydrins, non-reducing sugars, sucrose, reactive oxygen species (ROS) scavenging, as well as switching off of metabolism, have been so far postulated as playing major roles in this phenomenon [5]. It has also been suggested that DT demands structural stabilizing processes, such as plasmalemma displacement and intracellular space vacuolization [6], [7]. In short, the mechanisms involved in DT may be roughly divided in three groups: 1) signalling mechanisms, gene regulation and functional proteomics; 2) metabolic adjustment and antioxidant systems; and 3) macromolecular and mechanical stability [8].

Several studies of the acquisition of DT during seed development [4], [9] and on its loss upon germination [10], [11], [12], [13] have been reported. Interestingly, DT can be rescued in germinated seeds by the application of a mild osmotic stress. The re-induction of DT in germinated seeds by incubation in PEG was first reported by Bruggink and van der Toorn (1995). They showed that DT could be fully restored in germinated seeds of *Cucumis sativus* and *Impatiens walleriana*. These authors suggested that this approach could serve as a convenient model system in studies of DT and may have important implications for the agricultural industry [14]. This strategy to re-induce DT in germinated seeds has been confirmed in other species like Medicago (*Medicago truncatula*) and *Tabebuia impetiginosa*. The re-establishment of DT in primary roots of Medicago germinated seeds by a mild osmotic stress (-1.5 MPa) treatment has been so far used to identify the transcriptome and (heat stable) proteome associated with DT [11], [15]. Furthermore, the application of a cold or heat shock prior to osmotic treatment improved desiccation tolerance in protruded radicles of *T. impetiginosa* [16]. These findings suggest the existence of overlapping mechanisms acting in parallel, or synergistically, in different stress types. Thus, understanding DT would not only enhance insights related to tolerance mechanisms of water deficit but also to other stresses, such as cold, salt and heat.

Despite numerous studies on the acquisition of DT during seed development and the re-induction of DT in germinated seeds, none of these have been able to ‘filter out’ the mechanisms that are not directly associated with DT but are coupled with other concomitant developmental pathways. Therefore, models capable of discriminating between overlapping developmental programs and the acquisition of DT are extremely promising to understand the genetic and molecular mechanisms controlling desiccation tolerance and sensitivity in seeds.

Arabidopsis (*Arabidopsis thaliana*) is a well-known model system in plant biology. Evidently, the use of this system together with all the genetic and molecular tools so far developed would generate a powerful model to further unravel the regulation and mechanisms of DT in higher plants. However it is not known whether germinated seeds of Arabidopsis are desiccation sensitive and if DT can be re-established after it has been lost upon completion of germination.

Here we show that Arabidopsis seeds lost DT upon germination and that DT can be re-induced in germinated seeds. Further we present the associated transcriptome of desiccation sensitive (DS) and DT germinated Arabidopsis seeds. The discovery of relevant genes may bring new insights to identify new strategies for crop production under abiotic stresses and highlight putative key hubs involved in the regulation of seed survival in the dry state. Furthermore, the use of Arabidopsis for studying loss and reestablishment of DT in germinated seeds in combination with genetic and molecular tools developed for this model species, engenders a powerful model to further unravel DT in higher plants.

## Materials and Methods

### Assessment of desiccation tolerance

**Figure 1:**
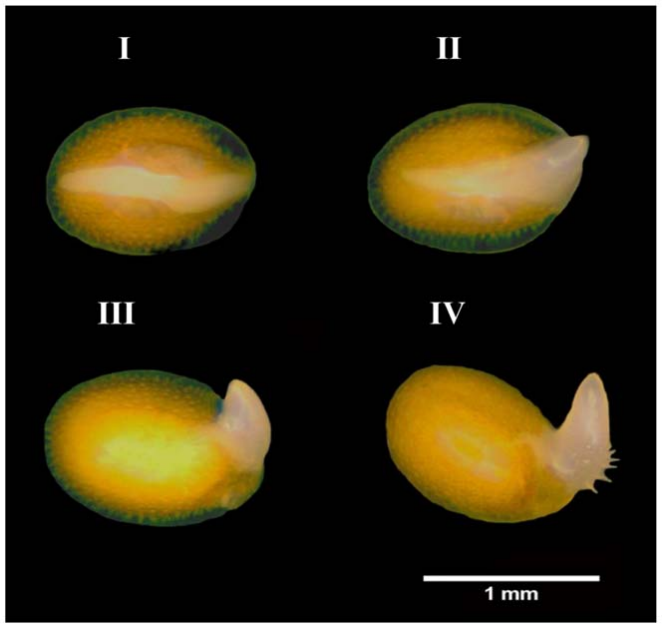
Arabidopsis seeds at different developmental stages during and after visible germination. I - testa rupture; II - at radical protrusion; primary root of approximately 0.3 mm length; and IV – at appearance of the first root hairs.

Seeds of Arabidopsis, accession Columbia (Col-0), were cold stratified for 72 h at 4°C in 9 cm Petri dishes on two layers of blue filter paper (Anchor paper Co.) and 10 ml of distilled water. After stratification to break residual dormancy, germination was performed at 22°C under constant white light and determined from three independent replicates of 100 seeds, by counting the number of individual seeds that had a protruded radicle. To determine the percentage of desiccation-tolerant germinated seeds, four developmental stages were defined. For that a stereomicroscope was used and the seeds were grouped as follows: (stage I) testa rupture; (stage II) seeds at radical protrusion; (stage III) germinated seeds showing a primary root of 0.3–0.5 mm length; and (stage IV) at the appearance of the first root hairs (Figure 1). These developmental stages occurred approximately 24, 28, 32 and 36 hours after the seeds were transferred from 4°C to the optimum germination conditions at 22°C. Four replicates of 25 seeds for each stage were fast-dried for three days at 20°C under a forced air flow at 32% relative humidity (RH), which was achieved by a saturated calcium chloride solution in a closed chamber. Water contents were assessed gravimetrically for triplicate samples of 70 germinated seeds, by determination of the fresh weight and subsequent dry weight after 17 h at 105°C [17]. Water contents were expressed on a dry weight basis. After dehydration, germinated seeds were pre-humidified in humid air (100% RH) for 24 h at 22°C in the dark, in order to avoid imbibitional damage [18], and then rehydrated in H2O at 22°C on a Copenhagen Table under a 12/12 h dark/light regime. Germinated seeds that continued their development and transformed into viable seedlings were considered desiccation-tolerant.

### Dehydration curves

Cold-stratified seeds were placed to germinate at 22°C under constant white light. Three replicates of 70 germinated seeds of the four developmental stages (Figure 1) were selected, placed in small aluminium pans and dried under a saturated CaCl_2_ atmosphere inside a drying chamber with a forced air flow (32% RH at 20°C). Concomitantly, three replicates of 70 germinated seeds of each developmental stage were picked-up and incubated in 6-cm petri dishes containing 1.2 ml of a polyethylene glycol (PEG 8000) solution with an osmotic potential of -2.5 MPa on one layer of filter paper at 22°C. After 3 d of PEG incubation and a quick wash in distilled water to remove residual PEG, the seeds were transferred to small aluminium pans and dried under a saturated CaCl_2_ atmosphere inside a drying chamber with a forced air flow. During the drying step and PEG incubation, samples were taken at intervals to measure water content by gravimetry.

### Assessment of the loss and re-establishment of DT

To assess the re-establishment of DT in germinated seeds, they were selected by their developmental stage (I, II, III and IV – Figure 1) using a stereomicroscope and either (fast) dried directly or after 3 d of incubation in PEG solution. Incubation was done in the dark at 22°C, in 6-cm Petri dishes containing 1.2 ml of PEG solution (-2.5 MPa) on one sheet of filter paper. After incubation, germinated seeds were rinsed thoroughly in distilled water with the aid of a set of sieves, transferred to new Petri dishes with one dry sheet of germination paper and then dehydrated, pre-humidified and rehydrated as described before. Germinated seeds that resumed growth and generated a viable seedling after rehydration were considered DT. Four independent experiments of 25 germinated seeds each were carried out for each treatment.

### RNA extraction, target synthesis and microarray hybridization

Germinated seeds of stage II after PEG incubation (DT) and non-treated germinated seeds at the same developmental stage (DS) were used for the RNA extractions. Total RNA was extracted according to the hot borate protocol modified from Wan and Wilkins (1994) [19]. Three replicates of approximately 1000 germinated seeds for each treatment were homogenized and mixed with 800 µL of extraction buffer (0.2M Na borate decahydrate (Borax), 30 mM EGTA, 1% SDS, 1% Na deoxycholate (Na-DOC)) containing 1.6 mg DTT and 48 mg PVP40 which had been heated to 80°C. 1 mg proteinase K was added to this suspension and incubated for 15 min at 42°C. After adding 64 µl of 2 M KCL the samples were incubated on ice for 30 min and subsequently centrifuged for 20 min at 12,000 g. Ice-cold 8 M LiCl was added to the supernatant in a final concentration of 2 M and the tubes were incubated overnight on ice. After centrifugation for 20 min at 12,000 g at 4°C, the pellets were washed with 750 µl ice-cold 2 M LiCl. The samples were centrifuged for 10 min at 10,000 g at 4°C and the pellets were re-suspended in 100 µl DEPC treated water. The samples were phenol chloroform extracted, DNAse treated (RQ1 DNase, Promega) and further purified with RNEasy spin columns (Qiagen) following the manufacturer's instructions. RNA quality and concentration were assessed by agarose gel electrophoresis and UV spectrophotometry. RNA was processed for use on *Affymetrix®* Arabidopsis SNPtile array (atSNPtilx520433) as described by the manufacturer. Briefly, 1 µg of total RNA was reverse transcribed using a T7-Oligo(dT) Promoter Primer in the first-strand cDNA synthesis reaction. Following RNase H-mediated second-strand cDNA synthesis, the double-stranded cDNA was purified and served as template in the subsequent *in vitro* transcription (IVT) reaction. The IVT reaction was carried out in the presence of T7 RNA Polymerase and a biotinylated nucleotide analog/ribonucleotide mix for complementary RNA (cRNA) amplification and biotin labeling. The biotinylated cRNA targets were then cleaned up, fragmented, and hybridized to the SNPtile array. The hybridization data was extracted using an R-script with the help of an annotation-file based on TAIR9 annotation (http://aquilegia.uchicago.edu/naturalvariation/cisTrans/ArrayAnnotation.html). Data were normalized in R using quantile normalization and average results for the 3 arrays per sample were used for further analysis. All data are MIAME compliant as detailed on the MGED Society website http://www.mged.org/Workgroups/MIAME/miame.html and the data discussed in this publication have been deposited in NCBI's Gene Expression Omnibus [20] and are accessible through GEO Series accession number GSE30853 (http://www.ncbi.nlm.nih.gov/geo/query/acc.cgi?acc=GSE30853).

### Microarray analysis

For analysis of the DT/DS gene set, germinated seeds after PEG treatment versus non-treated germinated seeds at the same developmental stage, we used the over-representation analysis (ORA) tool of GeneTrailExpress [21]. This analysis was employed to identify significantly enriched gene ontologies (GO) categories. ORA was performed with the following parameters: significance level: 0.05, p-value adjustment for multiple testing: Bonferroni adjustment, minimum class size: 3, maximum class size: 40.

To identify cis-acting promoter elements potentially involved in regulating the co-expression of genes involved in DT, the Arabidopsis expression network analysis (*Athena*) tool was used (http://www.bioinformatics2.wsu.edu/cgi-bin/Athena/cgi/home.pl) [22]. Specifically, the promoter regions of all differentially up-regulated genes (Fold change ≥2 and P-value ≤0.05) were searched for any common motifs located within a 1 kb region upstream of the translational start site.

## Results

### Assessment of the re-establishment of desiccation tolerance

**Figure 2:**
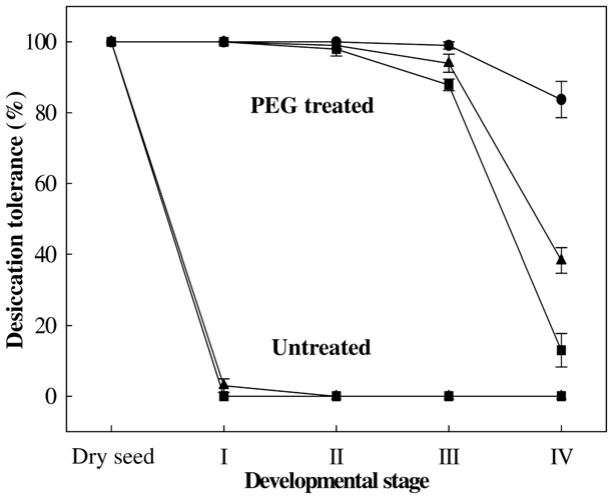
Re-establishment of desiccation tolerance in Arabidopsis. Desiccation tolerance was determined after drying the germinated seeds with or without previous PEG treatment, followed by pre-humidification and rehydration. Survival of cotyledons (circles) and primary roots (squares) was scored 5 d after rehydration and seedlings (triangles) 10 d after rehydration. Each data point is the average of four independent experiments of 25 seed/seedlings. Bars represent standard error.

Previous reports have shown that DT can be fully rescued in germinated seeds [10], [14], [16]. Here it was tested whether DT could be re-induced in Arabidopsis seeds by treating them in a PEG (-2.5 MPa) solution for three days at 22°C. DT can be re-induced in imbibed seeds only in a limited time frame and its loss usually coincides, depending on the species, with the protrusion of the radicle tip and/or radicle length [10], [14], [16]. Therefore we defined four clearly distinct developmental stages to assess for re-induction of DT. The stages are: stage I (testa rupture), stage II (seeds at radical protrusion), stage III (germinated seeds showing a primary root of 0.3–0.5 mm length) and stage IV (at the appearance of the first root hairs) (Figure 1). First we determined the best developmental stage suitable for the re-establishment of DT. For all stages DT could be re-established to a certain extent however seeds at earlier stages (i.e. stages I and II) performed better as compared to the two later stages. Seeds completing germination at stage I and germinated at stage II, showed 100% of re-establishment of DT regarding primary roots, cotyledons and seedling formation while for those at stage III a slight reduction in all parameters was observed (Figure 2). Germinated seeds at stage IV showed low competence in re-establishing DT with a final survival rate of 38% (Figure 2). This treatment pointed to a developmental stage-dependent re-establishment of DT and, in addition, indicates that different seed parts have differential sensitivity to drying. Interestingly, more seedlings survived due to lateral root formation. This phenomenon was also observed by several other authors. They frequently noted the appearance of lateral roots after the primary root had been lethally damaged by desiccation [14], [16], [23]. Seedlings that did not resume radicle growth, frequently showed growth of the cotyledons and, to a lesser extent, also of the hypocotyl. However, the longer the radicle before dehydration, the less frequent the growth of cotyledons and hypocotyl.

**Figure 3:**
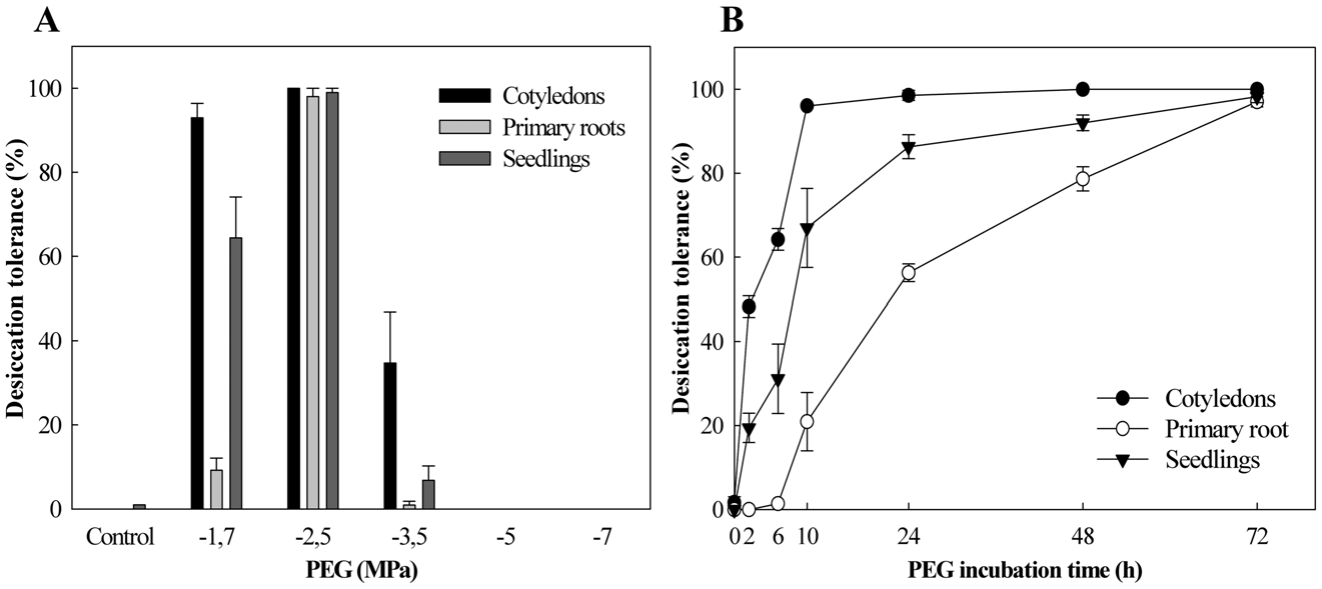
Osmotic potential and incubation time effects on re-establishment of desiccation tolerance. (A) PEG osmotic potential effect on DT; (B) -2.5 MPa PEG solution incubation time effect on DT. Survival of cotyledons and primary roots was scored 5 d after rehydration and seedlings 10 d after rehydration. Each data point is the average of four independent experiments of 25 seeds at developmental stage II. Bars represent standard error. Control  =  germinated seeds dried directly at 32% RH without previous PEG treatment.

Next we investigated the effect of the osmotic potential and time of incubation on the ability to re-establish DT in germinated Arabidopsis seeds. Therefore, first, seeds at stage II were submitted to different PEG concentrations (Figure 3A). DT could be substantially re-induced (approximately 100%) for all parameters in seeds treated with PEG -2.5 MPa. A lower PEG osmotic potential (-1.7 MPa) re-induced DT as well and resulted in nearly 100% survival of the cotyledons after drying. However, this concentration resulted in a huge drop of primary root survival to approximately 10%. A larger percentage of seedlings survived (60%) although this depended on lateral root formation. From -2.5 MPa downwards there was an abrupt drop in DT re-establishment, decreasing to 34% for cotyledons, 1% for primary roots and 7% for seedling formation at -3.5 MPa, to 0% for all parameters at -5.0 and -7.0 MPa (Figure 3A).

Thus a concentration of -2.5 MPa PEG solution is the optimal concentration to re-establish DT of stage II Arabidopsis seeds. Lastly we varied the incubation time from several hours up to three days using stage II seeds in a -2.5 MPa PEG solution. As the acquisition of DT is an active process [24], the incubation time also appeared to be crucial to the recovery of DT. A minimum of three days in -2.5 MPa PEG was necessary for full re-establishment of this attribute in Arabidopsis germinated seeds (Figure 3B).

### Drying responses

**Figure 4:**
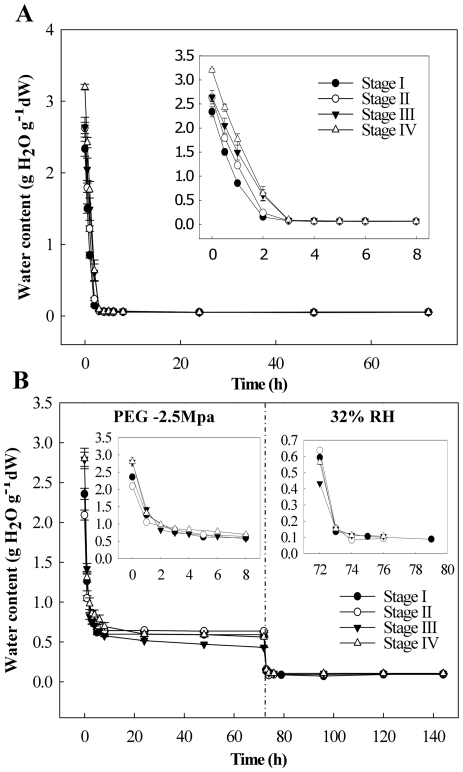
Arabidopsis seed water content changes upon dehydration. Dehydration at 32% RH (A) and incubation in PEG followed by dehydration at 32% RH (B). Inserts show the first hours of drying in more detail. Bars represent standard error.

Different dehydration conditions, especially drying rates, can significantly influence the response of desiccation-sensitive plant tissues [25]. Therefore we first studied the de-hydration behaviour of the Arabidopsis seeds at all four defined stages. All developmental stages showed similar behaviour in relation to the drying procedures and achieved water content (WC) levels as low as 0.08 g H_2_O g^−1^ dry weight by the end of the first 6 hours of drying (Figure 4A). The drying responses of germinated seeds incubated in PEG -2.5 MPa (optimal treatment, see below) was characterized (Figure 4B). All the developmental stages responded similarly when exposed to PEG and further drying at 32% RH. Despite a small difference in initial WC of seeds in the different stages, with the highest in the most advanced stages, they achieved similar WCs by the end of the PEG incubation (0.5 g H_2_O g^−1^ dry weight) and after CaCl_2_ drying (0.05 g H_2_O g^−1^ dry weight) (Figure 4A and B).

### The Arabidopsis transcriptome in desiccation sensitive and -tolerant germinated seeds

**Figure 5:**
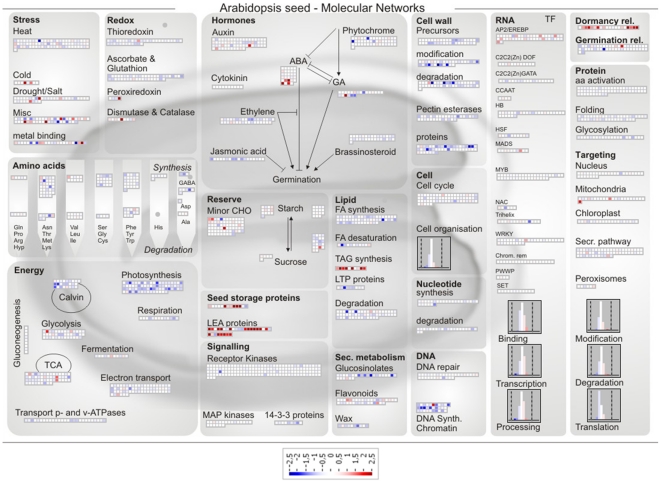
Seed MapMan molecular network map. Log2 ratios are used to express relative transcript levels in germinated Arabidopsis seeds at stage II treated for 3 d in PEG -2.5 MPa versus non-treated seeds in the same developmental stage. Red squares, higher levels in PEG treated seeds; blue squares, higher levels in non-treated seeds. Only ratios with P values lower or equal to 0.05 are displayed.

Our next step was to carry out a global microarray analysis to identify the genes whose expression changed in the germinated seeds at stage II after PEG incubation (DT) in comparison with that of the non-treated germinated seeds at the same developmental stage (DS). To catalogue genes whose expression responded to the PEG treatment with confidence a cut-off based filter was applied. Genes were selected if their expression exhibited at least 2-fold enhancement/reduction in expression after three days of incubation in PEG in comparison with the non-treated samples, and if the enhancement/reduction in expression was statistically significant (P-value ≤0.05) over three independent biological replicates. The array used in this experiment covers 30.509 genes in the Arabidopsis genome and the application of this filter resulted in a list (DT/DS gene set) of 677 genes, of which 263 were up-regulated and 414 were down-regulated (Table S1). These genes exhibited an unclustered distribution across the range of hybridization intensities, which indicates an unbiased representation of gene expression. In order to visualize the overall differentially expressed genes (P-value ≤0.05), we used the Page-Man/MapMan package (http://MapMan.gabipd.org). This tool allows users to display genomic datasets onto pictographic diagrams to get a global overview of the ontology of the up- and down-regulated genes [26]. We used the seed-specific MapMan pathway, which efficiently captures the most relevant molecular processes in seeds [27]. In DT germinated seeds, up-regulation was found of transcripts encoding for cold and drought stress responsive genes, LEA (Late Embryogenesis Abundant) proteins, seed storage proteins, such as cruciferins and PAP85, enzymes involved in triacylglycerol synthesis, transcription factors, especially those interacting with drought and ethylene responsive elements, and dormancy related proteins (Figure 5 and Table S1). Furthermore, genes encoding for ABA signal transduction elements and ABA biosynthesis were up-regulated, concomitantly with the down-regulation of GA biosynthetic genes such as *AtGA3ox2* and GA-responsive genes like AGPs (arabinogalactan proteins) and *GASA3*. Genes related to carbohydrate breakdown and antioxidant activity were also triggered in the DT germinated seeds (Figure 5). In contrast, a massive repression of genes related to DNA biosynthesis and chromatin structure, energy metabolism and cell wall modification was seen in the DT germinated seeds (Figure 5). Regarding the ‘Energy’ class, down-regulation occurred particularly for genes related to photosynthesis and the Calvin cycle (Figure 5).

**Figure 6:**
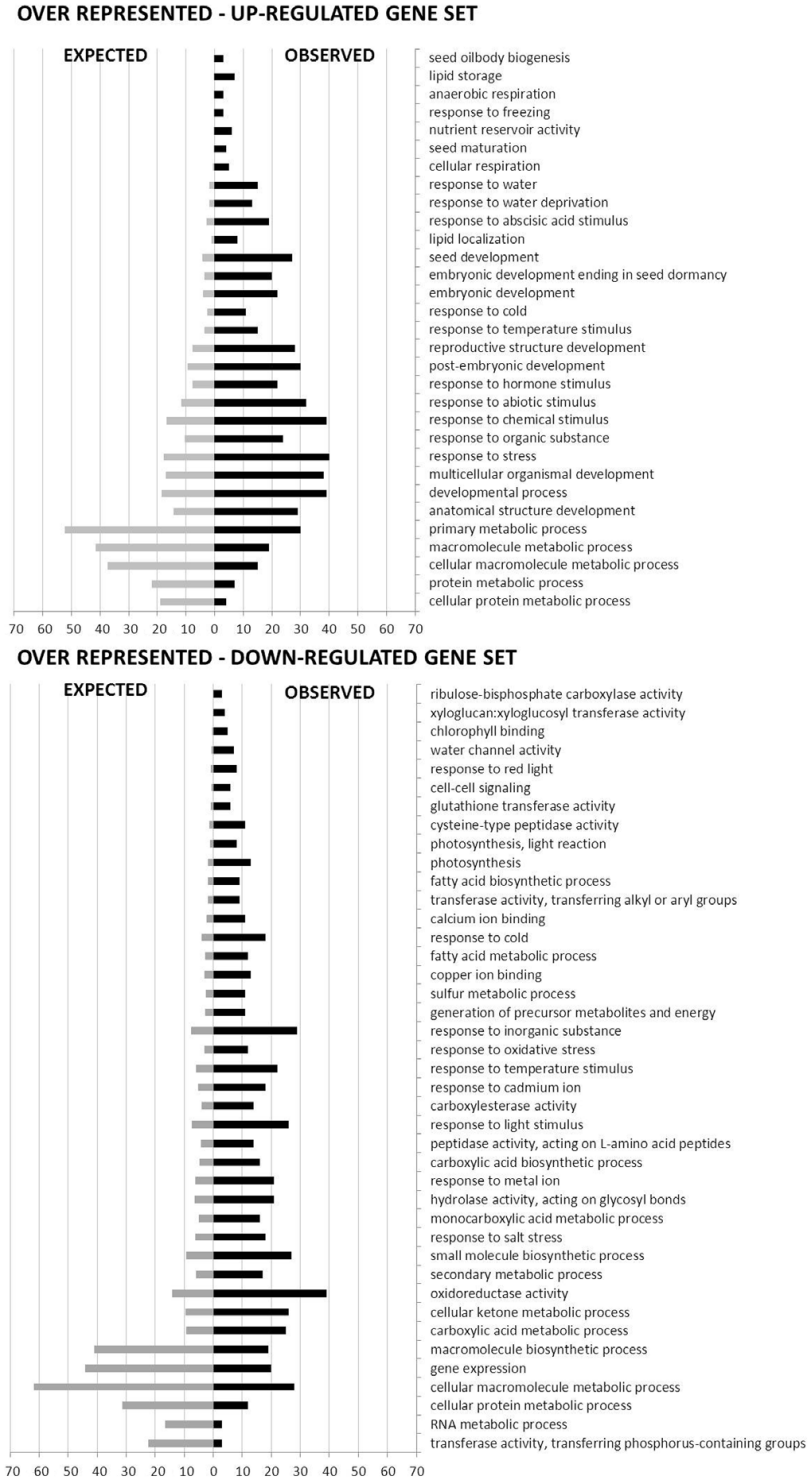
Over-representation analysis of the differentially expressed genes after PEG treatment. The gene set analyzed was first filtered through a fold-change and variance cut-off based filter (fold change ≥2 and P-value ≤0.05). The bars are listed in relation to the observed/expected genes ratio (high to low).

In order to verify whether our strategy to visualize and filter the gene set was valid, a more detailed analysis was undertaken. We used the over-representation analysis (ORA) tool of GeneTrailExpress [21]. This analysis compares a gene set of interest to the reference set and when considering a certain functional category as a gene ontology (GO) term, it attempts to detect if this category is over-represented or under-represented in the respective gene set. It also estimates how likely this is due to chance [21]. The filtered gene set was split into two subsets, up- and down-regulated, and the program allowed us to determine which GO categories were significantly enriched (P-value ≤0.05) in the DT/DS up- and down-regulated gene subsets (Figure 6). GO terms describing developmentally related processes such as ‘lipid storage’, ‘nutrient reservoir activity’ and ‘seed maturation’ together with GO terms describing responses to various abiotic stresses responses such as freezing, water deprivation and cold were among the top-ranked enriched processes in the DT/DS up-regulated gene subset. It is important to stress that the genes were ranked in relation to the observed/expected ratio and GO terms such as the ones related to seed development, embryonic development ending in seed dormancy, post-embryonic development, response to hormone and abiotic stimulus as well as response to stress were represented by a large number of genes in the DT/DS up-regulated gene subset (Figure 6). Furthermore, response to water deprivation and abscisic acid (ABA) stimulus categories were, at the same time, represented by a high number of genes and high-ranked, reinforcing the importance of the hormone ABA to the acquisition of DT. Drought responsive genes such as *DREB2A, XERO1*, LEA genes and ABA-responsive genes, such as *EM1, GEA6, RAB18, LTI65, RD29B*, among others, appeared in the DT/DS gene set (Table S1). Interestingly, the *RD29B* gene, which was highly up-regulated in the DT/DS gene set (Table S1), was not listed in the GO category ‘response to ABA stimulus’ (Table S2), showing that the power of this analysis can be limited by the GO annotation's accuracy. *LTI65* contains two ABA-responsive elements (ABREs) that are required for the dehydration-responsive expression of *RD29B* as cis-acting elements [28]. On the other hand, all the drought responsive genes that were highly up-regulated in the DT/DS set such as *PER1, RAB18, ATDI21* and *DREB2A*, were retrieved in the GO category ‘response to water deprivation’. Analyses of *DREB2A* has shown that it is possible to increase drought stress tolerance of the transgenic plants overexpressing this gene and revealed that DREB2A regulates the expression of many water stress inducible genes [29], [30]. As expected, GO categories denoting processes related to energy such as ‘response to red light’, ‘chlorophyll binding’ and ‘photosynthesis’, as well as GO categories grouping genes related to cell wall breakdown and loosening, metabolism of fatty acids, among other post-germination related processes, were over-represented in the down-regulated DT/DS gene subset. For example, xyloglucan endotransglycosylases/hydrolase (XET) genes, such as *MERI5B, XTH9* and *TCH4* were down-regulated in the DT/DS gene set (Table S1). This gene family encodes for enzymes that modify a major structural component of the plant cell wall, xyloglucans, and therefore may influence plant growth and development [31].

**Figure 7:**
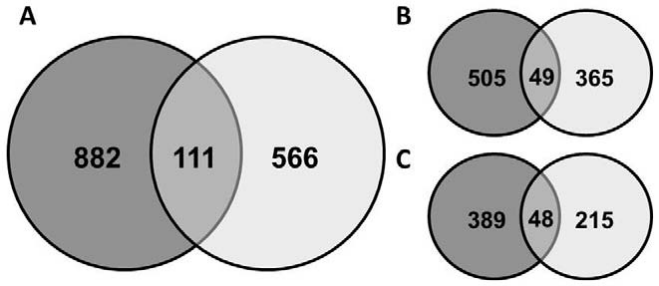
Overlapping homologous genes in Arabidopsis and Medicago germinated seeds after re-establishment of DT. (A) total number of overlapping genes; (B) down-regulated in both systems and; (C) up-regulated in both systems. The gene lists are presented in Table S2.

**Figure 8:**
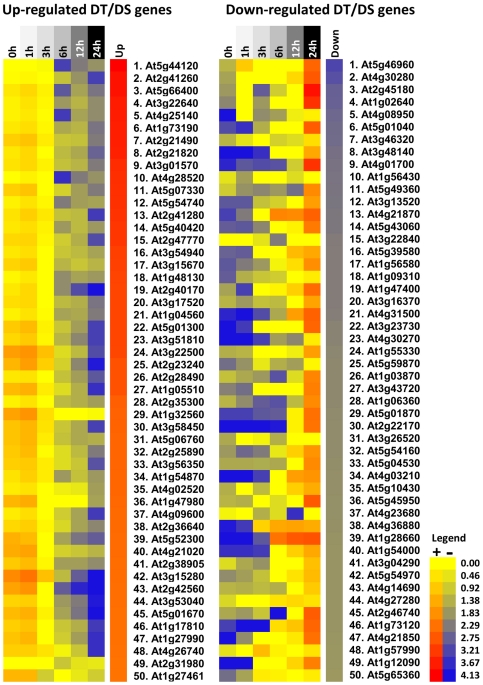
Heat maps displaying the gene expression profile during seed imbibition according to the Bio-Array Resource database. The top 50 up- and down-regulated genes in the DT/DS set were tested, in silico, for their expression during seed imbibition (0 to 24 h). The DT/DS up-regulated genes showed up-regulation (red) in dry seeds and down-regulation (blue) during seed imbibition. The DT/DS down-regulated genes showed down regulation (blue) in the dry seed and in early phases of seed imbibition and up-regulation along germination. The values in the legend are log2-transformed ratios.

Furthermore, we compared the gene list after the cut-off filter (DT/DS gene set - Table S1) against a data set obtained in a similar system for Medicago [11] and a significant overlap was found. Among 111 genes that were present in both Arabidopsis and Medicago gene sets, 49 were down- and 48 were up-regulated in both species and 14 displayed opposite response (Figure 7 and Table S3). As in Arabidopsis, genes encoding for antioxidant activity, ABA signalling, seed storage proteins, LEA proteins, drought and ethylene responsive elements and dormancy related traits were up-regulated in the rescued Medicago germinated seeds (Table S3, Figure S1 and Table S4). The most significant overlap in the down-regulated genes occurred for genes related to cell wall and energy metabolism. The genes showing opposite response belonged to a varied range of classes. Based on these results we hypothesized that a controlled reversion from the germination program towards the seed developmental program is taking place during the incubation in PEG and that this transition is necessary for the re-establishment of DT. To verify this, we examined the expression during seed imbibition of the top 50 ranked of both up- and down-regulated genes in the DT/DS set and created a heat map in the Expression Browser of the BAR website [32]. The heat maps clearly corroborated our hypothesis and it appears indeed that the germinating seeds are reverted to a developmental, desiccation tolerant, stage (Figure 8). According to this *in silico* analysis, genes that were up-regulated in the PEG treated seeds and may be related to the acquisition of DT were down-regulated upon imbibition while genes that were down-regulated in the treated seeds were up-regulated upon imbibition (Figure 8). More details about this analysis can be seen in the supplementary material (Table S5).

### Identification of promoter motifs within up-regulated enriched genes

Information about the promoter region of genes can provide valuable information on how these genes are regulated. Besides that, the presence of similar elements in different genes can suggests common regulatory mechanisms of their expression. An analysis of the differentially up-regulated genes in the DT/DS gene set was performed to identify potential *cis*-acting promoter elements. In particular, this analysis identified two main transcription factor (TF) site groups overrepresented within the promoters of germinated seeds treated with PEG. The first group consists of 11 enriched motifs containing the core sequence (ACGTG), named ABA-responsive element (ABRE) and the second of 2 enriched motifs containing the drought responsive elements (DRE), core motif (A/GCCGACA). Next to these two groups also a MYC-related, RY element and an Evening element were overrepresented. Table 1 displays the promoter element consensus sequence, number of promoters with TF sites and the number of predicted TF sites.

**Table 1:** Most common *cis*-acting promoter elements within the most differentially up-regulated genes in Arabidopsis germinated seeds at stage II treated with PEG^a^.

TF sites containing ABRE-motif	Consensus sequence b	P	S	P-value
ABFs biding site motif	CACGTGGCACGTGGC	36	41	<10^−10^
AtMYC2 BS in RD22	CACATGACATG	102	142	<10^−4^
ABRE-like biding site motif	BACGTGKMACGTGKM	137	275	<10^−10^
ACGTABREMOTIFA2OSEM	ACGTGKCCGTGKC	128	217	<10^−10^
CACGTGMOTIF	CACGTGACGTG	104	302	<10^−10^
GADOWNAT	ACGTGTCCGTGTC	88	129	<10^−10^
GBOXLERBCS	MCACGTGGCCACGTGGC	32	36	<10^−10^
ABRE binding site motif	YACGTGGCACGTGGC	55	64	<10^−10^
ABREATRD22	RYACGTGGYRYACGTGGYR	34	37	<10^−10^
GBF1/2/3 BS in ADH1	CCACGTGGCACGTGG	14	28	<10^−5^
Z-box promoter motif	ATACGTGTTACGTGT	20	20	<10^−6^
**TF sites containing DRE-motif**				
DREB1A/CBF3	RCCGACNTCCGACNT	38	46	<10^−7^
DRE core motif	RCCGACCCGAC	91	122	<10^−10^
**Other TF sites**				
MYCATERD1	CATGTGATGTG	102	142	<10^−4^
RY-repeat promoter motif	CATGCATGATGCATG	19	38	<10^−4^
Evening Element promoter motif	AAAATATCTAAATATCT	32	38	<10^−4^

a Analysis performed with *Athena* [21].

b Where B = T/G/C; K = T/G; M = A/C; Y = T/C; R = A/G and N = A/T/G/C.

The number of promoters containing at least one instance of the TF binding site and the total number of TF binding sites in the selected set of sequences are given in the ‘P’ and ‘S’ columns, respectively. The p-value for enrichment is a measure of how overrepresented a motif is in the selected set of genes versus the overall occurrence in the genome.

## Discussion

The ability to study desiccation tolerance/sensitivity with a combination of ‘omics’ techniques and *in vivo* physiology, creates new opportunities for examining how organisms deal with desiccation stress. Here we explore the possibility to rescue desiccation tolerance in desiccation sensitive, germinated, Arabidopsis seeds by PEG-treatment and its associated transcriptome. By observing changes in gene expression in germinated seeds of Arabidopsis in response to the PEG treatment, this study aimed at giving insights into the metabolic and regulatory changes necessary to induce DT. It also introduces the model of re-establishment of DT in Arabidopsis germinated seeds as a valuable tool to unravel this trait. To date several laboratories have shown the capacity of re-establishing desiccation tolerance in germinated desiccation-sensitive seeds, such as cucumber and *Impatiens* [14], *Medicago truncatula* [10] and *Tabebuia impetiginosa* [16]. However, to our knowledge, this is the first report of such an approach for Arabidopsis seeds.

In our experimental conditions (drying at 32% RH at 20°C), DT was completely lost before radicle protrusion (stage I) and could be completely rescued at stages I and II. DT is commonly lost before visible germination. However, for some species it can be rescued until certain developmental stages after visible germination (radicle protrusion). Evidently, even for an apparently homogeneous batch of seeds, germination does not occur uniformly. Consequently, at any given point of the germination time course, the population of seeds is comprised of germinated and non-germinated seeds of different developmental stages. Thus, to characterize the loss and re-establishment of DT in a more precise way, we defined four developmental stages to assess this trait. The precise definition of developmental stages reduced heterogeneity within and between biological replicates and increased experimental reproducibility. The use of precisely defined developmental stages (Figure 1) also brings up the possibility to investigate why DT can be rescued, after it is lost, only in a short developmental frame between germination and seedling establishment. As observed for other species, [3], [10], [23], [33], the different Arabidopsis embryonic tissues displayed distinct sensitivities to drying and progressively lost tolerance to desiccation upon radicle protrusion. The fact that we were able to rescue DT in seeds that were incubated in PEG at 22°C, makes this system in Arabidopsis more robust in relation to the ones so far reported. For example, it avoids possibly overlapping responses associated with cold, since in all other reports the combination of an osmotic stress and low temperature was used to rescue DT in sensitive germinated seeds [10], [14], [16].

Our genome-wide analysis of the genes that responded to the PEG treatment identified a total of 263 genes as being up-regulated and 414 genes as being down-regulated. The functional analysis of this data set suggests that mechanisms related to proteins, DNA and membrane stabilization, such as accumulation of soluble sugars and LEA proteins [10], [34] aided by activation of antioxidant and ROS scavenging systems [5], [8], [11] were required for the re-establishment of DT (Figures 5 and 6). Our data supports the notion that to re-establish DT, Arabidopsis germinated seeds partially return to a quiescent stage prior to germination which resembles the dry seed (Figure 8). In agreement with this, several developmental and dormancy related genes were re-activated (Figures 5 and 6) and promoter motifs related to abiotic stresses, but also to the dry seed stage, such as DRE, ABRE as well as MYCATERD1 and the RY-repeat containing motifs, were enriched within the promoters of up-regulated genes after PEG treatment (Table 1). One remarkable feature of the DT/DS gene set expression profile was that transcripts related to energy metabolism such as the ones encoding for components of the photosynthetic apparatus were among the most strongly down-regulated. One explanation is that metabolic processes such as photosynthesis and carbohydrate metabolism are sensitive to water deficit and plants use different strategies such as reduction of the photosynthetic rate and/or accumulation of protective molecules to avoid damage by ROS generated by photosynthesis [8]. One noteworthy example of such a strategy is employed by poikilochlorophyllous resurrection plants. These organisms break down chlorophyll and dismantle thylakoid membranes during dehydration in order to avoid ROS formation and it has been argued that this mechanism may be conserved in both vegetative tissues of resurrection plants and orthodox seeds [4], [35].

According to our data, the re-establishment of DT in germinated Arabidopsis seeds is comparable to the acquisition of DT in a range of different organisms. For instance, it is possible to find resemblances between the transcriptomes associated with DT in seeds, resurrection plants, mosses, fungi as well as with the acquisition of DT in developing seeds [35], [36], [37], [38]. As in those organisms, germinated Arabidopsis seeds experienced a complex cascade of molecular events including a combination of activation/deactivation of genes followed by biochemical alterations that lead to the acquisition of DT. Consistent with this idea, we found a considerable overlap, both in the up- and down-regulated genes when comparing the A. thaliana DT/DS gene set with the one from a similar experiment in Medicago [11]. Interestingly, the deactivation of photosynthesis seems to be less relevant to Medicago than to Arabidopsis. While a massive repression of photosynthesis related genes occurred in Arabidopsis, this was not observed in Medicago (Figure 5, Figure S1 and Table S4). Furthermore, besides being present in both systems, accumulation of transcripts related to sucrose and triacylglycerol (TAG) biosynthesis appears to have distinct relevance depending on the species. While in Arabidopsis the synthesis of TAG appears to be more relevant, the synthesis of sucrose seems to be more important for Medicago. The accumulation of sucrose and TAG has been described as playing protective roles in seeds and embryos [10], [14], [39]. This suggests that the observed differences could be due to intrinsic properties of those seeds. For example, Arabidopsis produces relatively more oleaginous seeds while Medicago produces starchy seeds. Furthermore the fact that there were more differences than similarities when comparing the gene sets of Medicago and Arabidopsis will partly be the result of differences in experimental set-up and used material. In our experiment RNA was extracted from whole Arabidopsis seeds while only the radical tips were used in the case of Medicago [11]. Here we describe a robust physiological model in Arabidopsis seeds that can mimic other DT systems and may contribute to a comprehensive understanding of stresses associated with desiccation tolerance and sensitivity by utilizing the evident advantages of this species, such as the extensive mutant collections. Comparing the available data sets related to DT in different organisms could pinpoint the core mechanisms that are orchestrating DT and are conserved among multiple organisms. In contrast, the differences in the expression of genes between these two species point that during DT establishment, the fundamental processes required are likely to be universal, although the causal individual genes are not necessarily conserved, and more species-specific.

## Supporting Information

Figure S1 **Seed MapMan molecular network map of** ***Medicago truncatula*** **DT/DS gene set (data from** [**11**] **, expression values in Table S4).** Log2 ratios are used to express relative transcript levels in germinated (3 mm long radicles) *Medicago truncatula* seeds treated for 3d in PEG -1.7MPa in relation to non-treated seeds in the same developmental stage. Red squares, higher levels in PEG treated seeds; blue squares, higher levels in non-treated seeds. Only ratios with P-values lower or equal to 0.05 are displayed.
(TIF)

Table S1 **Differentially up- and down-regulated genes (DT/DS gene set) after the re-establishment of desiccation tolerance (DT) in germinated Arabidopsis seeds at stage II by PEG treatment.**
(XLS)

Table S2 **Over-representation analysis of the differentially expressed genes after PEG treatment.** The analyzed gene set was first filtered through a fold-change and variance cut-off based filter (fold change ≥2 and P-value ≤0.05).
(XLS)

Table S3 **Comparison between the Arabidopsis gene list for expression after re-establishment of desiccation tolerance (DT/DS gene set - Table S1) against a data set obtained in a similar system for Medicago** [**10**] **.**
(XLS)

Table S4 **Log2 ratios of relative transcript levels in germinated (3-mm long radicles)** ***Medicago truncatula*** **seeds treated for 3d in PEG -1.7MPa in relation to non-treated seeds in the same developmental stage with the respective AGI code of the closest Arabidopsis homologue.** Only ratios with P-values lower or equal to 0.05 are shown [11].
(XLS)

Table S5 **Details of the heat maps displaying the gene expression profile during seed imbibition according to the Bio-Array Resource database.** The top 50 up- and down-regulated genes in the DT/DS set were tested, in silico, for their expression during seed imbibition.
(XLS)
