# Supplementary figures and images for: The Re-Establishment of Desiccation Tolerance in Germinated Arabidopsis thaliana Seeds and Its Associated Transcriptome
Source: PLoS One. 2011 Dec 14;6(12):e29123. doi: 10.1371/journal.pone.0029123 (PMC3237594; doi:10.1371/journal.pone.0029123)

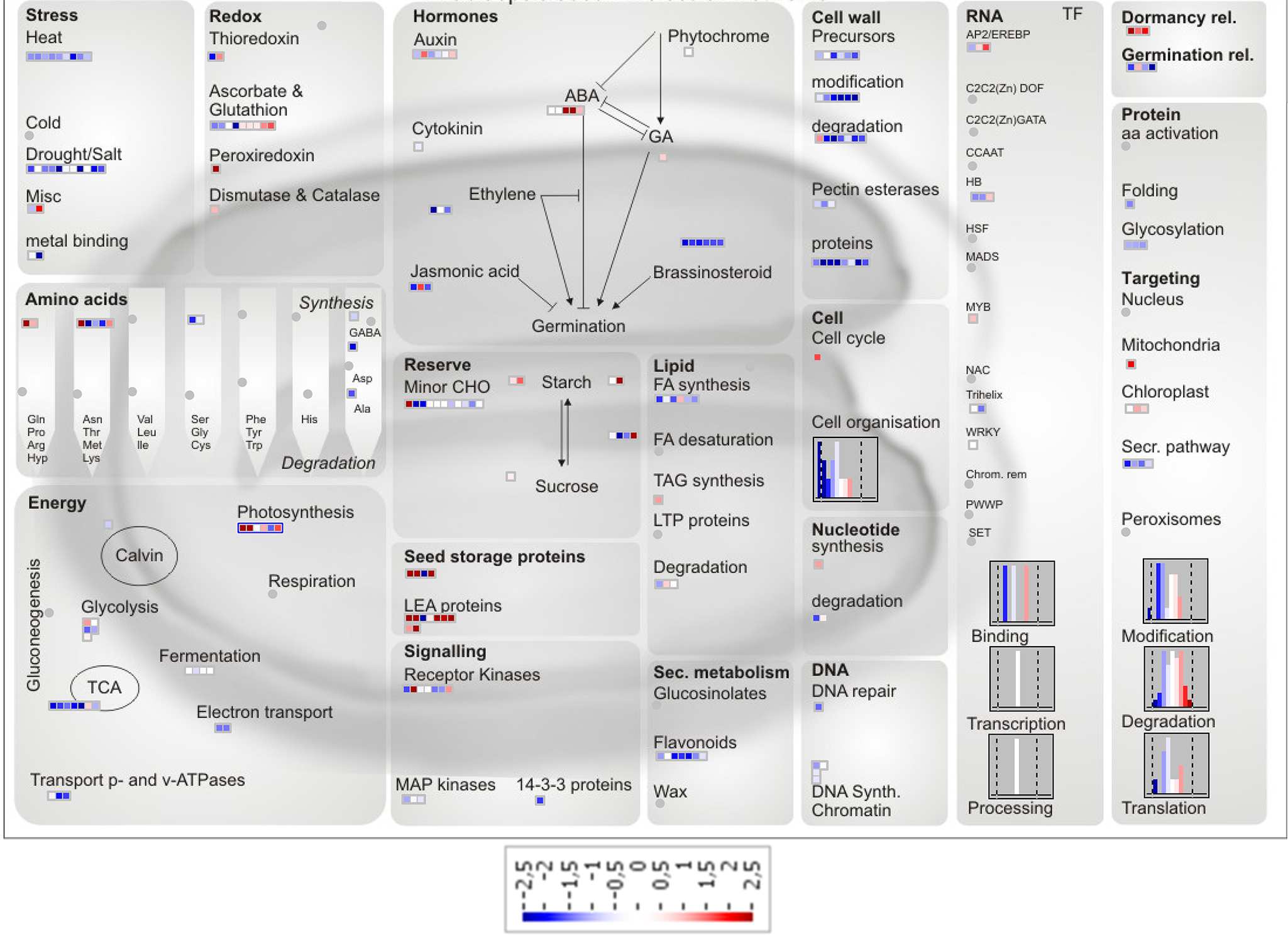

Supplement: Figure S1 — Seed MapMan molecular network map of Medicago truncatula DT/DS gene set (data from [11] , expression values in Table S4). Log2 ratios are used to express relative transcript levels in germinated (3 mm long radicles) Medicago truncatula seeds treated for 3d in PEG -1.7MPa in relation to non-treated seeds in the same developmental stage. Red squares, higher levels in PEG treated seeds; blue squares, higher levels in non-treated seeds. Only ratios with P-values lower or equal to 0.05 are displayed. (TIF) [file pone.0029123.s001.tif]
